# Effects of offloading devices on static and dynamic balance in patients with diabetic peripheral neuropathy: A systematic review

**DOI:** 10.1007/s11154-020-09619-9

**Published:** 2021-01-16

**Authors:** Koen Andre Horstink, Lucas Henricus Vincentius van der Woude, Juha Markus Hijmans

**Affiliations:** 1 Center for Human Movement Science, University of Groningen, University Medical Center Groningen, Groningen, The Netherlands; 2 Center for Rehabilitation, University of Groningen, University Medical Center Groningen, Groningen, The Netherlands; 3 School of Sport, Exercise & Health, Peter Harrison Centre for Disability Sport, Loughborough University, Loughborough, UK; 4 Department of Rehabilitation Medicine, University of Groningen, University Medical Center Groningen, Groningen, The Netherlands

**Keywords:** Neuropathy, Offloading, Balance

## Abstract

Patients with diabetic peripheral neuropathy (DPN) usually have reduced somatosensory information and altered perception in feet and ankles. Somatosensory information acts as feedback for movement control and loss of somatosensation leads to altered plantar pressure patterns during gait and stance. Offloading devices are used to reduce peak plantar pressure and prevent diabetic foot ulcers. However, offloading devices can unfortunately have negative effects on static and dynamic balance. It is important to investigate these unwanted effects, since patient with DPN already are at high risk of falling and offloading devices could potentially increase this risk. The aim of this systematic review is to investigate the effects of plantar offloading devices used for ulcer prevention on their role in static and dynamic balance control in patients with DPN. PubMed and Embase were systematically searched using relevant search terms. After title selection, abstract selection, and full-text selection only five articles could be included for further analysis. Two articles included static balance measurements, two articles included dynamic balance measurements, and one article included both. Results suggested that static balance control is reduced when rocker bottom shoes and different insole configurations are used, however, toe-only rockers showed less evidence for reduced static balance control. There was no evidence for reduced dynamic balance control in combination with offloading devices. However, these results should be interpreted with care, since the number of studies was very small and the quality of the studies was moderate. Future research should evaluate balance in combination with different offloading devices, so that clinicians subscribing them are more aware of their potential unwanted consequences.

## Introduction

Diabetes mellitus (DM) is a major problem worldwide, especially among elderly. Diabetic patients are prone to develop neuropathy at their feet and ankles [1]. The incidence of diabetic peripheral neuropathy (DPN) among newly diagnosed diabetic patients is approximately 30% [2]. This increases to approximately 50% in patients diagnosed with DM for more than 20 years [3]. DPN is characterized by functional loss of cutaneous receptors and proprioceptive sensation, also referred to as somatosensory loss [4]. Loss of somatosensory information indicates reduced perception, which starts most often in feet and lower legs of DM patients, and subsequently, may lead to foot ulcers [1]. High plantar foot pressure and shear stress in combination with high blood sugar levels increases risk of developing diabetic foot ulcers (DFU) [5]. One in four diabetic patients develops DFU, which can have serious consequences and may lead to an amputation or even death [1]. Consequently, prevention of DFU through reduction of peak plantar pressure and shear stress during standing and walking is important in patients with DPN.

Older people with DM experience higher fall risks compared to healthy older people [6]. When accompanied by DPN fall risk increases even further. The central nervous system uses somatosensory information for maintaining balance in static and dynamic situations, such as bipedal balance control, which is evidently compromised in patients with DPN [7, 8]. Therefore, patients with DPN are at higher risk of falling compared to healthy older adults [9]. Other studies in healthy people found similar balance reductions as patients with DPN, e.g. when the feet of healthy people were placed in an ice bath [4]. This temporarily decreases somatosensory information from the feet. Besides reduced balance in patients with DPN, offloading devices such as insoles or diabetic footwear influence pressure distribution, afferent somatosensory information, and perception in patients with DPN [10, 11], which may also negatively influence balance control in patients with DPN.

Offloading devices are commonly used to prevent DFU occurrence or recurrence [12, 13]. There are many different devices used for plantar offloading and most of them aim at altering plantar pressure through foot positioning, roll-off characteristics, cushioning, and increasing foot surface support [12]*.* Offloading devices have different sole characteristics than regular footwear (e.g. thickness, stiffness, rocker position, base of support), which can have other positive or negative side effects. For example, shoes that consist of thicker midsoles and smaller base of support lead to decreased control of the centre of pressure, and thus, decreased postural stability [14]. In addition, decreased centre of pressure control results from decreased reactivity characteristics of the shoes or insoles on the foot surface. Research in healthy older people showed that postural stability decreases when standing on materials with low resiliency [15]. Besides centre of pressure control, shear stress can also have effects on balance control. Shear stresses are related to development of DFU and some offloading devices implement shear stress reducing techniques in their designs, for example shear stress reducing insoles [16]. These insoles permit lateral motion in the device to reduce shear stress at the skin and deeper tissues. However, side-to-side motion might cause patients with DPN to become unsteady [16]. On the contrary, other insole designs might be beneficial for balance control, since textured insoles have some beneficial effects in increasing somatosensory information and perception in patients with DPN, and subsequently increase postural stability [17].

All in all, the exact relation between plantar offloading in diabetic patients and balance control in diabetic patients is not clear, although both received much attention separately. It is important to examine this relation, since most patients with DPN are already at higher risk of falling [6, 18]. The aim of this systematic review is to investigate the effects of plantar offloading devices used for prevention of DFU on static and dynamic balance control in patients with DPN. It is expected that offloading techniques negatively influence static and dynamic balance control, and consequently, can lead to higher fall risks in patients with DPN.

## Methods

### Search strategy

The electronic databases PubMed and Embase were systematically searched. Combinations of free text words ‘balance’, ‘posture’, ‘stability’, ‘gait’, ‘equilibrium’, ‘fall’, ‘offloading’, ‘pressure’, ‘redistribution’, ‘distribution’, ‘neuropathy’, ‘polyneuropathy’, ‘diabetes’, ‘plantar’, ‘foot’, and ‘feet’ were used in combination with OR and AND Booleans. In addition, Mesh terms ‘Postural balance’, ‘Posture’, and ‘Diabetic neuropathies’ were used in PubMed. Corresponding Emtree terms ‘Body equilibrium’, ‘Body Position’, and ‘Diabetic neuropathy’ were used in Embase. Detailed search terms are shown in Appendix 1. Search strategies were applied at 8 January 2020 and no time period restrictions were used.

### Study selection

After removing duplicates, paper selection followed a three-step approach. Firstly, title screening was performed by two reviewers (K.A. Horstink & J.M. Hijmans). For initial selection based on title, the following criteria were used: (1) title should contain ‘offloading’ (or equivalent) in combination with an offloading device; or (2) the title includes ‘balance’ (or equivalent) and the title refers to ‘diabetic neuropathy’ (or equivalent). Exclusion of articles based on title was done (1) if the article was a review; (2) if the study population concerned participants with an amputation; or (3) if the text was in another language than English or Dutch.

Secondly, the abstracts of the remaining papers were further scanned, again by the same two reviewers. Abstract selection was performed using the following additional inclusion criteria: (1) Studies should aim at diabetes or diabetic neuropathy; if healthy participants were used, a relation to foot and balance problems of patients with DPN should be made; (2) use of an offloading device for prevention of DFU; (3) at least one balance related outcome measurement should be reported; (4) the number of participants is equal or greater than 1; and (5) offloading abilities of the offloading device should be mentioned as outcome measurement or the offloading device used should be known to effectively offload plantar surface. Articles were excluded if one of the following exclusion criteria was met: 1) Use of plaster or equivalent devices that specifically restrict ankle motion; 2) studies that include patients with any kind of lower limb amputation; 3) studies that are not primary research; and 4) participants were younger than 18 years. Disagreement between reviewers was discussed during a consensus meeting and all disagreements were resolved.

Subsequently, full-text selection was performed by the two reviewers using the same inclusion criteria with the following additional criteria: (1) the study should contain at least one measurement outcome that is related to static balance or dynamic balance (or both); (2) only primary research is included that is published as full text; and (3) full text is written in English or Dutch. Exclusion criteria were the same as during the abstract selection. During a consensus meeting, disagreements between reviewers were discussed. When the two reviewers had no consensus about inclusion of papers, assessment by a third independent co-author (L.H.V. van der Woude) provided binding advice.

References used in the selected studies were checked to identify literature that was not found with the used search strategy. These studies were included in the selection if they met all in- and exclusion criteria as mentioned above

### Quality assessment

**Fig. 1:**
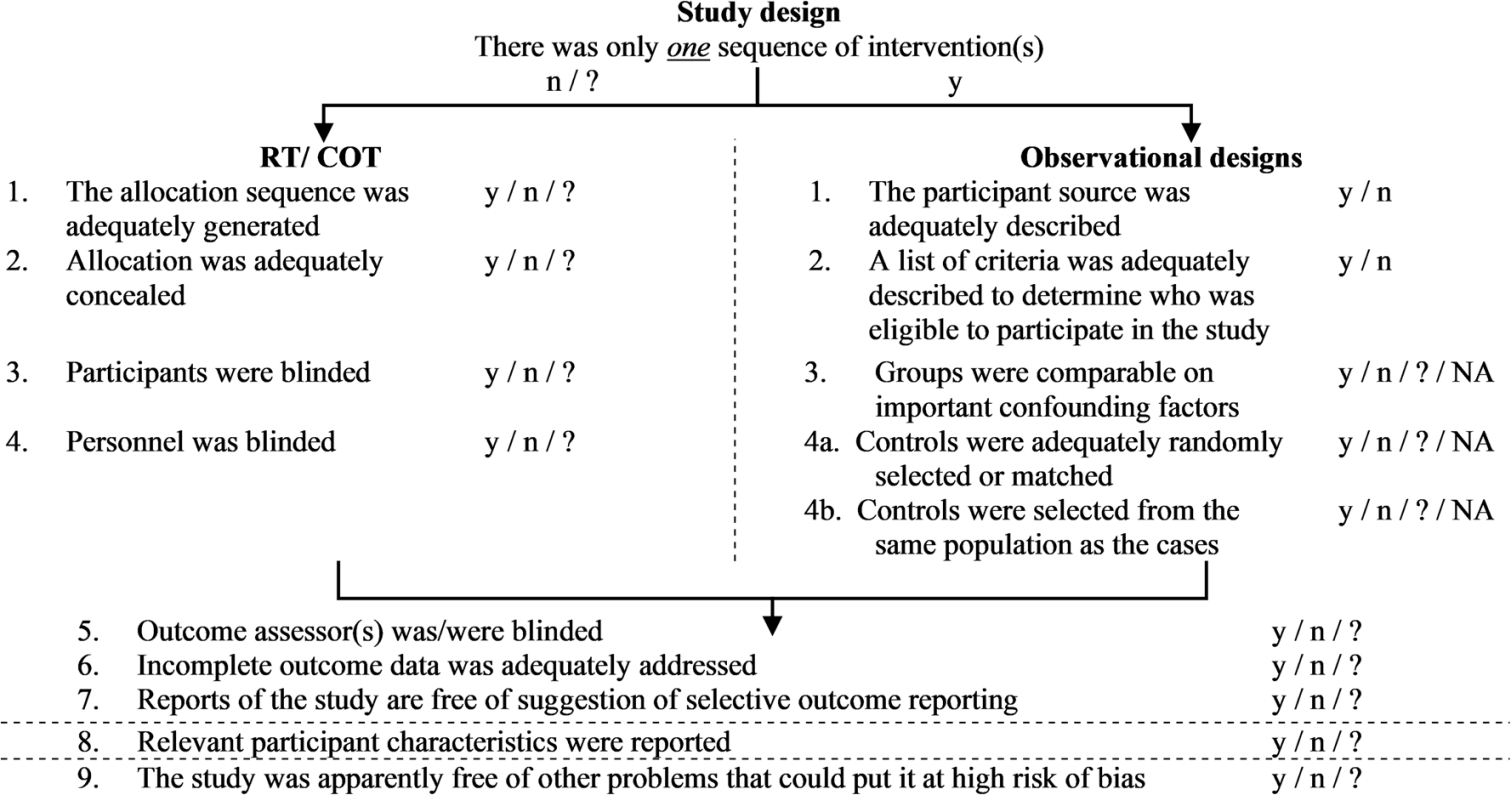
Quality assessment tool modified from van der Wilk et al. [19]. RT = randomized trial, COT = cross over trial, y = yes (low risk of bias), n = no (high risk of bias),? = unclear (uncertain risk of bias), NA = not applicable

Since studies of different designs and scientific quality could potentially be included, a general quality assessment scale was used which essentially is applicable to studies with different designs. Van der Wilk et al. [19] developed a quality assessment tool for similar purposes. In this study a modified version of Van der Wilk et al. [19] was used (Fig. 1). Van der Wilk et al. [19] based their quality assessment tool on the risk of bias for randomized controlled trials and cross-over trails [20], the PEDro scale [21], and the Wales list [22]. Judgment scores included ‘not applicable’, ‘low risk of bias’, ‘high risk of bias’, and ‘unclear risk of bias’. 1–3 ‘Yes’ answers were considered a high risk of bias, 4–6 ‘Yes’ answers were considered as moderate risk of bias, and 7–9 ‘Yes’ answers were considered as low risk of bias. Two reviewers (K.A. Horstink & J.M. Hijmans) assessed the quality of the included studies.

## Results

### Literature search

**Fig. 2:**
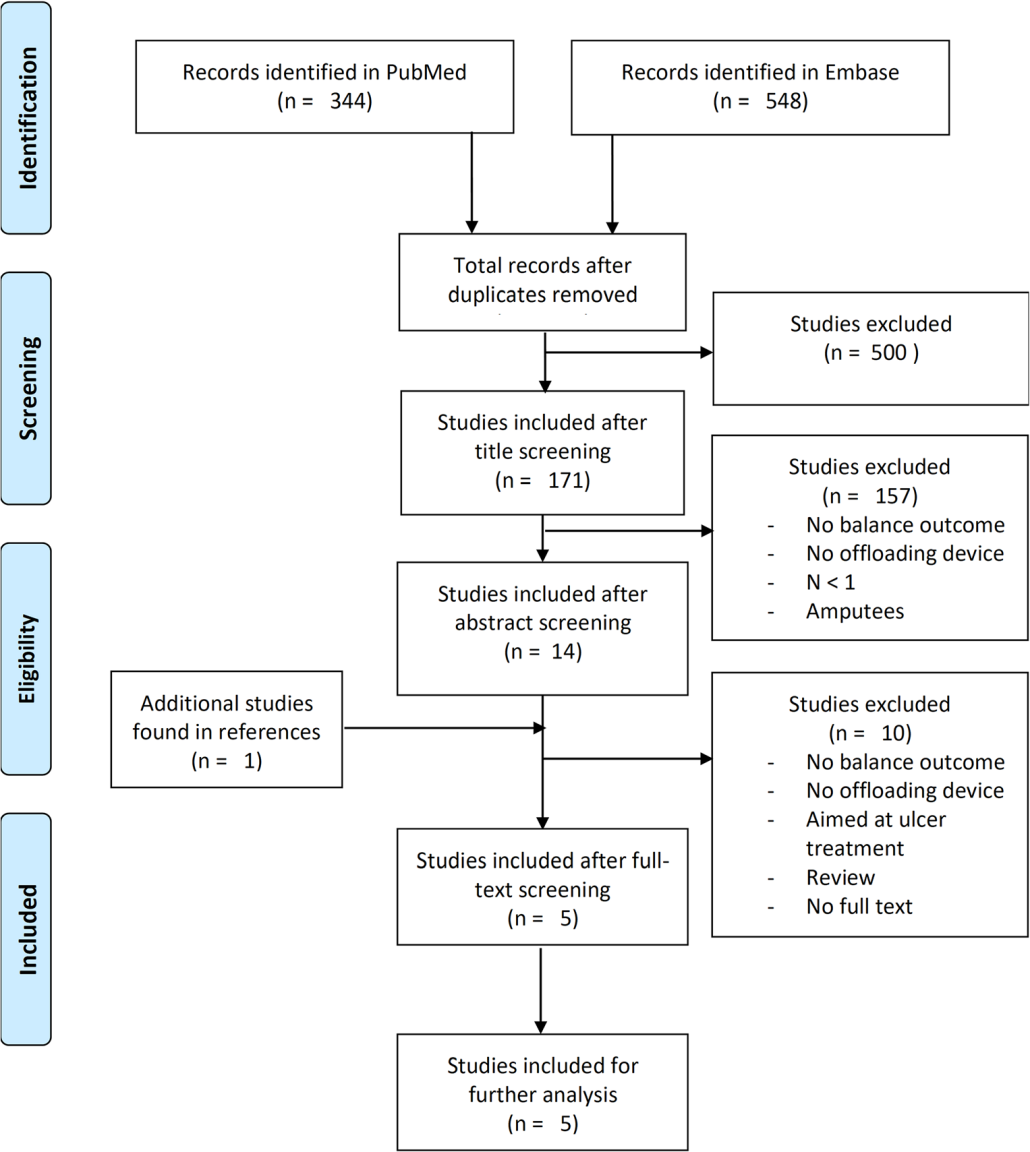
Flow-chart of the literature selection process, modified from the PRISMA statement [23]. The literature search was performed in PubMed and Embase

The search strategy resulted in 344 hits in PubMed and 548 hits in Embase. After removing duplicates 671 articles were identified. A flow chart of the selection process is presented in Fig. 2, the flow chart was modified from the PRISMA statement for writing a systematic review to fit the selection process used in this study [23]. Based on title selection 500 records were excluded. Abstract selection was performed on the remaining 171 articles. Subsequently, full text screening was performed on the remaining 14 articles and one article found in the references was added to the full text screening. Five articles were eventually included for detailed analysis (Fig. 2).

### Study quality

Following the quality assessment, Ghomian et al. [24, 25] and Paton et al. [17] had five ‘yes’ answers and the risk of bias was considered moderate. Albright et al. [26] with four ‘yes’ answers was also considered to have a moderate risk of bias. Grewald et al. [27] had only three ‘yes’ answers with a consequent high risk of bias (Table 1).

**Table 1:** Results of the quality assessment for the five included studies. The numbers correspond to the criteria as shown in in Fig. 1

Author	Reference	Observational design					General				
		1	2	3	4a	4b	5	6	7	8	9
Ghomian et al.	[24]	y	y	na	na	na	?	?	y	y	y
Ghomian et al.	[25]	y	y	n	n	?	?	?	y	y	y
Paton et al.	[17]	y	y	na	na	na	?	?	y	y	y
Albright et al.	[26]	y	y	na	na	na	n	?	y	n	y
Grewald et al.	[27]	n	y	?	n	?	?	?	y	y	n

*y* yes (low risk of bias), *n* no (high risk of bias),? unknown or no information (uncertain risk of bias)

### Interventions

**Fig. 3:**
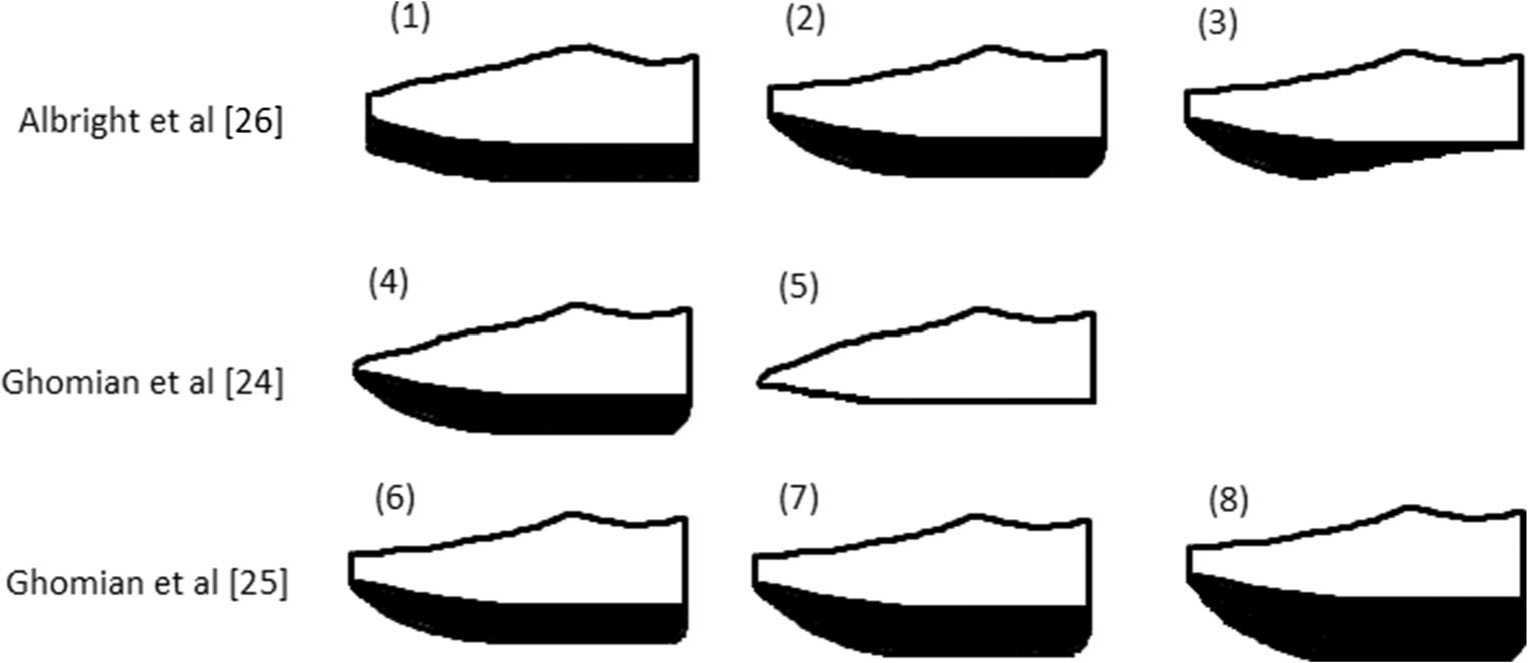
Schematic overview of the rocker bottom shoes used by Albright et al. [26] and Ghomian et al. [24, 25]. Numbers 1–3 correspond to the conditions used by Albright et al. [26], numbers 4 and 5 correspond to the conditions used by Ghomian et al. [24], and numbers 6–8 correspond to the conditions used by Ghomian et al. [25]. (1) = Control shoe; (2) rocker bottom shoe with rocker apex positioned at 60–65% of the shoe length; (3) negative heel shoe with rocker apex at 60–65% of the shoe length and from rocker apex to heel the sole was reduced to zero thickness; (4) rocker bottom shoe with rocker apex position at 62.5% of the shoe length and a rocker angle of 23°; (5) baseline shoe; (6) Rocker bottom shoe with rocker angle of 10°, apex position at 60% of the shoe length, and apex angle of 80°; (7) rocker bottom shoe with rocker angle of 15°, apex position at 55% of the shoe length, and apex angle of 80°; (8) rocker bottom shoe with rocker angle of 20°, apex position at 60% of the shoe length, and apex angle of 95°

Three of the five included articles used special shoes as offloading devices in their study [24–26]. Albright [26] et al. used rocker bottom shoes and negative heel shoes, which were modified from a normal shoe made of canvas with a rubber sole. Their control condition consisted of the unmodified shoe. Ghomian et al. [24] evaluated the effects of toe-only rockers and another study of Ghomian et al. [25] used different types of toe only rockers with different rocker angles of 10, 15, and 20 degrees. A schematic overview of the rocker bottom shoes used by Albright et al. [26] and Ghomian et al. [24, 25] is shown in Fig. 3. Paton et al. [17] used insoles with different characteristics as offloading device including standard diabetic insoles, insoles with removed arch fill, insoles with low resilient memory cover, and insoles with textured PVC cover. These different insole types were compared to the no insole control condition. Grewald et al. [27] used prescribed footwear of the included patients, which were compared with healthy participants. Two of the five articles included participants with diabetes or DPN [17, 24], two article studied patients with DPN and healthy people [25, 27], while one article included healthy young adults [26]. Table 2 provides an overview of the included studies with their characteristics, offloading devices, and results.

**Table 2:** Overview of the included studies with their characteristics and outcomes

Author	Design	N	Gender	Participants		Device	Control	Methods		Balance outcome measures	
(Ref)			(M/F)	Age (SD)	Type			Balance task	Acquisition	Static outcome measures	Dynamic outcome measures
Paton et al. [17]	Within-subject	50	38 M 12 F	71 (8.0)	DPN	SI RAI LRMI TI	No insole	Stance PS SRT	Force plate 1–10 scale In-shoe pressure	COPv ↑ 13.1%** LMRI vs CO; ↑ 14.2%* SI vs CO COPv ↑ 9.3%* LMRI vs TI; ↑ 9.9%* LMRI vs RAI COPl ↑ 13.2%** LMRI vs CO; ↑ 14.2%* SI vs CO COPl ↑ 9.4%* LMRI vs TI; ↑ 10.1%* LMRI vs RAI	No significant effects SRT No significant effects PS
Albright et al. [26]	Within-subject	20	3 M 16 F	Range: 22–25	H	RB NH	Control shoe	Stance with perturbation	Force plate	COMs ↑ 25.1%* RB vs CO; ↑ 28.6%* NH vs CO COMvar ↑ 19.3%* RB vs CO; ↑ 22.0%* NH vs CO COMapv ↓ 17.6%* RB vs CO; ↓ 20.0%* NH vs CO COMsr ↑ 9.5%* RB vs CO; ↑ 10.5* NH vs CO COMppv ↓11.9%ns RB vs CO;↓ 18.4%* NH vs CO COPs ↑ 15.0%* RB vs CO; ↑ 18.2%* NH vs CO COPvar ↑ 6.5%* RB vs CO; ↑ 8.6%* NH vs CO COPapv ↓ 13.9%* RB vs CO; ↓ 10.1%* NH vs CO COPppv ↓ 14.5%* RB vs CO; ↓ 11.7%* NH vs CO COPsr ↑ 0.1%ns RB vs CO; ↑ 0.0%ns NH vs CO FSM ↓ 23.7%* RB vs CO; ↓ 24.1%* NH vs CO	
Grewald et al. [27]	Between-subject	39	Unkown	54.2 (11.3) 58.3 (4.4) 59.6 (6.0)	15 DPN 16 DPNU 8 H	OF OS RCW HF	Healthy persons with HF	Walking 200 ft	Wearable sensors		DS ↑ 26.4%* DPN vs CO DS ↑ 6.3%ns DPNU vs CO DS ↑ 19.0%* DPN vs DPNU COMs ↓ 34%* DPN vs CO COMs ↓ 47%** DPNU vs CO COMs ↑ 23%ns DPN vs DPNU
Ghomian et al. [24]	Within-subject	17	7 M 7 F	49.3 (7.5)	DPN	TR	Control shoe	Stance with perturbation	Dual force plate	COFd mean all perturbations ↑ 6.2%ns TR vs GS RSS backward large perturbation ↓ 23.3%ns TR vs GS RSS mean other perturbations ↑ 24.5%* TR vs GS RL mean all perturbations ↓ 3.9%ns TR vs GS	
Ghomian et al. [25]	Between-subject	34	12 M 22 F	58.7 (4.7) 54.4 (5.1) 33.2 (2.0)	9 DPN 14 DM 11 H	TR10 TR15 TR20	Barefoot	10 m walking	Vicon		No significant effects SMOS^1^ FMOS↓ 6.2%ns B vs TR10^1^ FMOS ↓ 9.3%* B vs TR15^1^ FMOS ↓ 5.7%ns B vs TR20^1^ FMOS ↓ 2.9%ns TR10 vs TR15^1^ FMOS ↑ 0.5%ns TR10 vs TR20^1^ FMOS ↑ 3.4%ns TR15 vs TR20^1^

Ref = reference, Device = offloading device, N = number of participants, M = male, F = Female, H = healthy persons, DPNU = diabetic peripheral neuropathy with ulcers, DM = diabetic patients without neuropathy, SI = standard diabetic insole, RAI = insole with removed arch fill, LRMI = insole with low resilient memory cover, TI = insole with textured PVC cover, RB = rocker bottom shoes, NH = negative heel shoes, OF = offloading footwear, OS = offloading sandals, RCW = removable cast walker, HF = habitual footwear, FS = specific flexible shoes, RS = specific rigid shoes, US = usual footwear, TR = Toe-only Rocker, GS = gymnastic shoe, MF = midfoot, RF = rearfoot, CO = compared to control condition, PS = perceived stability, SRT = step reaction time, PDT = peak duration time, CoP = Centre of Pressure, CoM = Centre of Mass, COPv = CoP velocity, COPl = CoP path length, COPs = CoP sway amplitude, COPvar = CoP sway variance, COPapv = CoP anterior peak velocity, COPppv = CoP posterior peak velocity, COPsr = CoP sway range, COPsur = CoP surface, COMs = CoM sway amplitude, COMvar = CoM sway variance, COMapv = CoM anterior peak velocity, COMsr = CoM sway range, COMppv = CoM posterior peak velocity, COFd = centre of force displacement, RSS = response strength scale, RL = response latency, PDT = peak duration time, DS = double support stance, DPN = diabetic peripheral neuropathy, ML = medial-lateral and AP = anterior-posterior

^a^Only results of diabetic peripheral neuropathy patients are shown; * indicate a significant difference with *p* < 0.05; ** indicates a significant difference with *p* < 0.01; ns = non-significant

### Outcome measurements

Static balance outcomes were included in two studies [24, 26], dynamic balance outcomes were included by two studies [25, 27], and one study included both static and dynamic balance outcomes [17].

Albright et al. [26] measured static standing balance on a force plate with perturbation in healthy young adults. Centre of Pressure (CoP) parameters and the functional stability margin were recorded and used as outcome measures. The perturbation was a horizontal backward translation of the force plate (12.5 cm, 0.23 m/s, 0.55 s). Participants were asked to maintain balance without stepping while looking forward at an eye level target. Ghomian et al. [24] also used a force plate in combination with the Motor Control Test that consist of four horizontal platform perturbations (medium backward, medium forward, large backward, and large forward). Outcome measures were centre of force displacement, response strength scale, and response latency. Paton et al. [17] also measured static balance using a force plate and CoP was recorded during every trial. Participants were asked to close their eyes during the balance task.

Paton et al. [17] measured dynamic balance during a step reaction time test with an in-shoe pressure measurement system. Participants were instructed to step upon a step of 15 cm for every trial. In addition to dynamic balance, Paton et al. [17] included a measure of perceived stability. Participants were asked to rate overall perceived stability of the different conditions on a 1–10 scale, with 10 indicating most stable situation. Ghomian et al. [25] measured dynamic balance using a passive motion capture system. In all groups the participants were measured during walking. Measurement outcome included sagittal and frontal plane margin of stability. Grewald et al. [27] measured dynamic stability using wearable sensors. Participants were divided into one patient group with DPN, one patient group with DPN and an ulcer history, and one healthy group. They walked for 200 ft. over ground with their prescribed footwear at a self-selected speed. Centre of mass (CoM) sway and double support time were extracted from the wearable sensors.

### Intervention effects

Paton et al. [17] found that standard diabetic insoles and insoles with low resilient memory cover led to significant increases of CoP velocity compared to no insole. Standard diabetic insoles also led to significantly increases CoP path length compared to no insoles. Larger CoP velocity and larger CoP path length means decreased postural stability. On the contrary, insoles with removed arch fill and insoles with textured PVC cover did not result in significant differences compared to no insole condition. This indicates better postural stability compared to standard diabetic insoles and insoles with low resilient memory cover. The only differences between standard diabetic insoles and insoles with removed arch fill were that the standard diabetic insole had a moulded arch fill and heel cup. Textured insoles were found to have no differences compared to insoles with removed arch fill condition and the no insole condition. The main conclusion of the authors was that static balance is influenced by insoles and textured insoles counteract negative effects to a certain extend [17].

Regarding footwear, Albright et al. [26] found that CoP sway and CoP sway variance were significantly larger in rocker bottom shoes and negative heel shoes compared to the control shoes. Peak CoP velocity was significantly lower in both shoes compared to the control shoes. This indicated that both shoes showed negative effects on stability compared to the control condition. The authors concluded that both the rocker bottom shoe and the negative heel shoe destabilize perturbed stance and lead to increased imbalance potentials. Ghomian et al. [24] found no significant differences in centre of force displacement and response latency, indicating no negative effects on static balance. The response strength scale was significantly larger during all perturbations, except the large backward perturbation. The larger response strength scale indicated that the patient increased their muscular effort to maintain postural balance. A second possible explanation might be that the larger strength responses are due to the smaller base of support of the toe-only rocker [24].

Outcome measurements related to dynamic balance were used in three studies [17, 25, 27]. Paton et al. [17] found no differences in dynamic balance for different insole types. Paton et al. [17] used step reaction time as outcome measurement since stepping up a change in level is a common event that is related to falls [28]. Grewal et al. [27] measured gait characteristics and CoP during gait. CoM sway and double support time were significantly larger in patients with DPN with and without ulcers, indicating less stable gait pattern for both groups compared to healthy controls. However, participants in this study used their personal prescribed footwear. All participants used different offloading devices and the effects were averaged. As a result, the study outcomes cannot be used to assess balance effects of individual types of offloading devices. Ghomian et al. [25] found that patients with DPN had no differences in sagittal plane margin of stability between the different conditions. For the frontal plane margin of stability, patients with DPN showed significant reduction of the toe-only rocker with an rocker angle of 15 degrees compared to the barefoot condition. No other significant differences between the conditions were found for the frontal plane margin of stability (Table 2).

Paton et al. [17] was the only study that included measures of perceived stability. It is interesting to notice that perceived stability was not significantly different between the control group, DPN with ulcer group, and DPN without ulcers group. However, the outcome measures showed similar trends as the balance outcomes. The low resilient memory insole and the standard diabetic insole showed lower values for perceived stability compared to the other conditions.

## Discussion

### General summary

This systematic review aimed to study the effects of plantar offloading devices used for prevention of DFU on static and dynamic balance control. Included papers were limited in number and quality. Only five papers met all inclusion criteria and these were reviewed in detail. Three articles described offloading shoes, one article described the effects of different types of insoles, and the last article included several offloading devices (Table 1). Three studies found significant reductions of static or dynamic balance control in combination with offloading devices. One study found no significant effects of rigid and flexible shoes on balance control. Interestingly, of the three studies that included dynamic balance outcome measurements, two studies found no effects of offloading devices on dynamic balance control and one study found small positive effects of different footwear types on balance control. Perceived balance was included in one article as additional balance outcome measurement and no effects of insoles on perceived balance were found (Table 2).

### Insoles

Paton et al. [17] showed reduced static balance control of patients with DPN while wearing standard diabetic insoles and insoles with low resilient memory (relatively soft material). Balance was worse compared to no insole and the insole with removed arch fill. These results suggest that the moulded arch fill and heel cup induce postural instability in patients with DPN. Softness of insoles might compromise stability in patients with DPN since CoP control of healthy people is dependent on the rigidity of the material on which someone is standing. Several studies already found better CoP control in soft shoes compared to rigid shoes, suggesting the same results for soft insoles compared to rigid insoles [29, 30]. In addition, insoles with a moulded arch fills have a larger contact area with the foot. One possible explanation for the difference in balance might be that increased contact areas leads to decreased pressure, which decreases the somatosensory feedback, and consequently, decrease balance. On the other hand, research in healthy older adults shows that arch support insoles enhance standing balance and are beneficial for fall prevention [31], indicating a difference between patients with DPN and healthy older adults. Paton et al. [17] also found that added textured covers decrease negative effects of insoles on static balance. However, similar to the results from the removed arch fills, in healthy people it was found that textured insoles had no significant effect on static balance in eyes open and eyes closed conditions [32]. Reduced somatosensory information in patients with DPN might explain these differences with healthy people, because healthy older adults without neuropathy already have sufficient somatosensory information available for balance control [33]. Textured insoles and insoles with removed arch fill seem to stimulate somatosensory information, resulting in increased balance performance in patients with DPN. For that reason, patients with DPN may benefit more from stimulation of somatosensory information through the use of textured insoles or insoles with removed arch fill compared to healthy people [34].

Regarding dynamic balance, Paton et al. [17] found no differences in dynamic balance using different insoles. Other research found no negative effects of insoles or small positive effects of different types of insoles on dynamic balance [35, 36]. A reason for the difference between static and dynamic balance might be the sensory input during dynamic activities. Dynamical situations provide more somatosensory feedback from muscles, joints, ligaments, and other structures. This increase in somatosensory feedback results in reduced reliance on somatosensory information from the feet and an increased reliance on somatosensory information detected in other structures during dynamic balance control. This theory is supported by research on modelling human locomotion, where control of leg and foot joints requires afferent feedback from all lower leg structures [37]. It is also important to note that an increase in somatosensory information is not dependent on walking speed, since walking stability is the same for slow, normal and fast walking speeds [38, 39]. Thus, increased availability of information from other structures might be used as compensation for the loss of peripheral somatosensory information to prevent reductions in dynamic balance, independent of walking speed.

Dynamic balance is characterized by the ability to maintain a stable gait. This can be done by keeping the CoP within the base of support during dynamical activities such as walking. Measuring dynamic balance is challenging, since it consists of walking, turning, dual tasks, stepping over or on an object, and resisting perturbations. In the study of Paton et al. [17] the step reaction time test was used to evaluate dynamic balance. However, it can be argued that the step reaction time test does not reflect the whole concept of dynamic balance. Tests for dynamic stability should include multiple aspects to obtain overall dynamic balance results, since stepping is only one aspect of dynamic balance.

### Footwear

Albright et al. [26] found evidence that rocker bottom shoes decreased postural stability during perturbed stance in diabetic patients. In contrast, Ghomian et al. [24, 25] found no or little effects of toe-only rocker shoes, different toe-only rocker settings, and rigid or flexible shoes on postural stability respectively. Outcome differences between these studies can possibly be explained by the characteristics of the shoes. Rocker bottom shoes have reduced base of support. Base of support is an important factor in CoP control, and therefore, influences balance control in static situations [40]. In addition, stiffer soles are associated with faster and more reactive CoP control. In healthy populations evidence was found for decreased postural stability with increased softness of midsole [14, 41–43], decreased postural stability with thicker midsoles [14, 41], increased postural stability with high-collared shoes [42–44], and increased postural stability with increased base of support [45]. Diabetic patients often wear rocker shoes for ulcer prevention. Rocker shoes are characterized by thick and stiffer soles, apex positions around 55–60%, and, with that reduced base of support. Toe-only rockers, which are rocker shoes without a heel rocker, have a larger base of support compared to rocker shoes with a heel rocker. For that reason, rocker bottom shoes evidently have more negative effects on postural stability in patients with DPN compared with toe-only rockers and regular shoes with different rigidities.

In general, the number of studies conducted on dynamic balance in combination with offloading footwear is very small. In this systematic review only one study on dynamic balance in combination with offloading footwear was included and the study provided little to no evidence for negative nor positive effects on dynamic balance. It was found that different types of offloading footwear showed longer double support phase and less CoM sway during walking, which are no clear indications for impaired balance [27]. However, the design of the study was a three-group comparison and all participants wore their own prescribed footwear. Participants wore different types of footwear with varying mechanical characteristics (offloading footwear, offloading sandals, and removable cast walkers). As a result, effects of different footwear on balance could not be determined. For that reason, balance outcomes of the study were difficult to interpret and difficult to compare with other studies. Najafi et al. [46] investigated gait unsteadiness in patients with DPN during barefoot walking and walking with regular shoes. Results revealed gait unsteadiness in both conditions compared to healthy controls. It was also found that the addition of footwear improved gait steadiness in patients with DPN, indicating beneficial effects of footwear on dynamic balance.

### Other solutions

Besides textured insoles, vibrating insoles also stimulate proprioception through vibrations under the surface of the foot and they showed some beneficial effects in patients with DPN. Hijmans et al. [47] tested vibrating insoles on healthy participants and patients with DPN. In healthy participants no significant effects were found. In contrast, patients with DPN were found to significantly improve in balance during an attention demanding task [47]. Other research showed that vibrating insoles are also beneficial for healthy older adults, since it enhances somatosensory information and it improves balance in dynamic and static situations [48]. Vibrating insoles might be used in combination with offloading devices that lead to decreased balance, in such a way that patients have the offloading benefits of the offloading devices without compromising balance. In other recent research prototype footwear and insoles were developed to optimize gait in older adults [36]. The footwear consisted of a firm rubber sole (25 mm thick under heel and 18 mm under the forefoot), a high collar to support the ankle, a firm heel counter, a ten degree bevel in the heel, and a slip resisting outsole. The insole was textured with 4 mm ethyl vinyl acetate with dome shape projections. Results revealed better balance in healthy older adults [36]. These findings are interesting in the context of this review, however, it is not clear if the prototype footwear and insoles were also beneficial for offloading purposes. Both the vibrating insoles and the prototype devices have potential and future research is needed to evaluate their effects in patients with DPN.

As discussed before in this review, increased rigidity of shoes is associated with better CoP control [30]. Research on healthy participants suggest the same results for insoles, since evidence supports that rigid insoles had potential beneficial effects compared to soft insoles [49, 50]. Rigid insoles show less postural sway and CoP velocity. In addition, when visual feedback was removed postural sway in the soft insole increased, whereas the rigid condition showed better postural stability without visual feedback indicating decreased fall risk [49]. It is suggested that in patients with DPN rigid insoles have similar effect, while textured insoles and vibrating insoles also showed beneficial effects on balance in patients with DPN. It could be interesting for future research to combine one of these two with rigid insoles.

### Limitations & future research

A limitation of this systematic review is that only static balance and dynamic balance were included. Perceived balance was not included in the search terms, but can give more insight on the subjective side of balance in combination with offloading devices. In addition, fall risk and fear of falling were not included in this systematic review. This limits the results of the current study, since fall risk is an important factor in patient with DPN [9]. A systematic review of Mustapa et al. [51] found that postural instability and impaired balance during gait contributes to higher risk of falling in patients with DPN, providing evidence for a relation between fall risk and static and dynamic balance. Considering this relation, the results on static and dynamic balance found in this study can be seen as an indication of fall risk. However, more research is needed support this relation.

Secondly, this systematic review is aimed at offloading devices used for ulcer prevention. Offloading devices used for treatment might also have negative effects on static and dynamic balance control. Offloading devices used for ulcer healing, such as cast walkers, are mechanically different from offloading devices used for prevention. Therefore, specific conclusions about offloading devices used for treatment cannot be drawn using the result of this study. A further limitation of this systematic review is that only studies written in English and Dutch were included.

A major limitation was that measurement outcomes of dynamic balance control varied between the included studies. Static balance outcomes were aimed at CoP parameters in three included studies [17, 24, 26]. Even though not all studies used the same parameters, comparison between studies aimed at static balance was possible to some extent. In contrast, measurement outcomes of dynamic balance included step reaction time, weight shifting time, CoM parameters, and gait parameters. Consequently, results were difficult to compare between different studies. For future research it is important to have an overlapping framework used as guidance for measuring dynamic balance. For example, Buurke et al. [52] and the included study of Ghomian et al. [25] used the extrapolated CoM (XCoM) concept to identify dynamic stability, consisting of the CoM position with CoM velocity as additional component. Medio-lateral dynamic stability can be achieved by controlling the medio-lateral position of the XCoM relative to the base of support [53]. This concept is known as the margin of stability [54, 55]. The margin of stability is a good indication for dynamic stability and is easy to compare between subjects or studies. Thus, the XCoM and margin of stability are useful measurement outcomes in future studies that include dynamic balance.

The included articles investigated the effects of different offloading devices on static and dynamic balance. It is important to note that all studies aimed at short term effects of the offloading devices, since all of the studies used no familiarization with the device or a short time of familiarization up to fifteen minutes. Generalisation of the findings is not possible regarding the long term effects of offloading devices on static and dynamic balance and future research is necessary to investigate if these short term effect are also persistent in the long term.

Another important point is the need for awareness of balance problems in research settings and clinical settings and this has been addressed in this review. Loss of somatosensory information contributes to balance problems, which is known among clinicians and researchers in the area of patients with DPN [4, 8, 9, 11, 51, 56]. However, the potential imbalance effects of offloading devices has received little attention in research and following a review of Dixit et al. [56] it is not a part of clinical evaluation scenarios. Future research should focus on offloading devices such as footwear and insoles. To differentiate between different offloading devices (or different settings in offloading devices) a randomized within-subject measurement is the most optimal design. Measurement outcomes should include static balance, dynamic balance, and subjective balance measurements (i.e. balance confidence, perceived stability). As mentioned before, balance outcomes should be measured following similar protocols and measurement outcomes, which allows for comparison between different studies. Consequently, scientific evidence can make suggestions of the effect of offloading devices on static and dynamic balance to clinicians.

## Conclusion

Only five studies on the effects of offloading footwear on balance were found. The included studies showed negative effects of offloading devices on static balance control. Standard diabetic insoles, insoles with low resilient memory cover, rocker bottom shoes, and negative heel shoes showed negative effects on static balance. There was no evidence for negative effects on dynamic balance control. However, these results are based on a small body of evidence that investigated short term effects of footwear and insoles on balance. Thus, conclusions regarding change in balance control as a result of wearing offloading devices used for prevention of DFU should be interpreted with care.

More research is necessary to gain insight into the benefits and side-effects of offloading devices. Future research should focus on comparing different offloading devices or different settings of offloading devices using a within-subject design. Furthermore, studies should include measurement outcomes aimed at static balance control, dynamic balance control, and perceived stability on the short and long term.

## Data Availability

Not applicable.

## Appendix 1: Detailed search strategy

### *Search strategy PubMed*

Mesh terms:

1. Postural Balance
2. Posture
3. Diabetic neuropathies

*Search strategy:*

(“Postural Balance”[Mesh] OR Posture [Mesh] OR postur* [tiab] OR stability [tiab] OR balanc* [tiab] OR equilibrium [tiab] OR fall* [tiab] OR gait [tiab]) AND (offloading [tiab] OR pressure [tiab] OR redistrib* [tiab] OR distrib* [tiab]) AND (diabetic neuropathies [Mesh] OR neuropath* [tiab] OR polyneuropathy* [tiab] OR diabet* [tiab]) AND (foot [tiab] or feet [tiab] or plantar [tiab])

### *Search strategy Embase*

Emtree terms:

1. Body equilibrium
2. Body position
3. Diabetic neuropathy

*Search term:*

(‘body equilibrium’ OR ‘body position’ OR postur*:ab,ti OR stability:ab,ti OR balanc*:ab,ti OR equilibrium:ab,ti OR gait:ab,ti) AND (offloading:ab,ti OR pressure:ab,ti OR distribution:ab,ti OR redistrib*:ab,ti) AND (‘diabetic neuropahty’ OR neuropath*:ab,ti OR polyneuropathy*:ab,ti OR diabet*:ab,ti) AND (foot:ab,ti OR feet:ab,ti OR plantar:ab,ti)
